# Experimental Study of Photopolymer Resin Composition for AlN Ceramic 3D Printing via Digital Light Processing

**DOI:** 10.3390/polym17172344

**Published:** 2025-08-29

**Authors:** Ning Kuang, Yifan Liu, Wenjie Zhao, Junfei Wu

**Affiliations:** 1 College of Electromechanical Engineering, Qingdao University of Science and Technology, Qingdao 266061, China; kuang.ning@mails.qust.edu.cn (N.K.); fanhaofan1203@163.com (Y.L.); 2 College of Sino-German Science and Technology, Qingdao University of Science and Technology, Qingdao 266061, China

**Keywords:** 3D printing, additive manufacturing, DLP, aluminum nitride, resin composition

## Abstract

Aluminum nitride (AlN) ceramics exhibit exceptional properties that render them highly valuable for diverse industrial applications. However, conventional manufacturing techniques encounter significant challenges in fabricating complex AlN components with precise geometries. To address these limitations, digital light processing (DLP) has emerged as a promising additive manufacturing approach for AlN ceramics. This study presents a systematic investigation of the monomer composition in the photopolymer resin system through a comprehensive experimental evaluation. The results demonstrate that an optimized mixture of monomers ACMO (56.7 wt%), DEGDA (2.7 wt%), and TMPTA (40.6 wt%) yields photopolymer resin with superior comprehensive performance. Utilizing this optimized formulation, a 50 vol% solid loading AlN ceramic slurry was successfully prepared, and subsequently, dense AlN ceramic components were fabricated through DLP. This provides an important basis for optimizing the slurry preparation of AlN ceramic fabrication based on DLP 3D printing.

## 1. Introduction

Aluminum nitride (AlN) is a high-performance ceramic material widely recognized for its exceptional combination of properties, including high thermal conductivity, excellent electrical insulation, a low dielectric constant, chemical inertness, a low coefficient of thermal expansion, superior corrosion resistance, and outstanding mechanical strength [1,2]. Owing to these favorable characteristics, AlN has been extensively utilized across a broad range of industrial sectors, such as microelectronics, optoelectronics, energy systems, chemical processing, and transportation engineering [3].

Despite its growing industrial relevance, the fabrication of AlN ceramic components with complex geometries remains a significant challenge due to limitations inherent in conventional manufacturing techniques. Traditional ceramic forming methods, such as dry pressing, isostatic pressing, extrusion, injection molding, and gel casting, rely heavily on rigid molds, thereby restricting their suitability for producing parts with intricate internal features or freeform architectures [4,5,6,7,8]. Furthermore, conventional subtractive machining processes, including turning, milling, planing, grinding, and drilling, often result in the formation of microstructural defects such as microcracks, delamination, and, in extreme cases, catastrophic fracture [9]. These issues severely compromise the mechanical reliability and dimensional precision of the final components.

In light of these constraints, the development of advanced processing and forming technologies capable of producing high-performance, structurally complex AlN ceramics has emerged as a focal point in current materials science and engineering research. Particular attention has been directed toward novel techniques that offer mold-free fabrication, design flexibility, and reduced defect susceptibility, thus enabling the production of next-generation AlN components with enhanced structural and functional integrity.

Recent advances in rapid prototyping and manufacturing technologies have effectively mitigated the inherent limitations of conventional ceramic fabrication methods, enabling unprecedented design flexibility in the production of geometrically complex components [10,11]. Among these, additive manufacturing (AM), also referred to as 3D printing, has emerged as a transformative layer-by-layer fabrication approach that facilitates the direct conversion of digital models into physical objects [12]. AM offers several significant advantages over traditional methods, including faster production cycles, superior shaping precision, reduced material waste, and design freedom [13,14]. Ceramic additive manufacturing is broadly categorized into five primary techniques: laminated object manufacturing (LOM), fused deposition modeling (FDM) [15,16], selective laser sintering (SLS) [17,18], direct ink writing (DIW) [19,20], and photopolymerization-based methods. Of these, stereolithography (SL), liquid crystal display (LCD), and digital light processing (DLP) have gained prominence in the ceramic community due to their superior dimensional resolution, high surface finish quality, and scalability [21,22,23].

Photopolymerization refers to a process in which monomers, or oligomers, upon exposure to light, generate reactive species through photoinitiators, thereby initiating polymerization reactions to form a cross-linked network structure. A critical step in fabricating ceramics via photopolymerization-based 3D printing involves formulating a homogeneous ceramic-loaded photopolymer resin suspension. This process requires the careful incorporation of ceramic powder into a photopolymer resin system, supplemented with dispersants and other additives to maintain slurry stability. The photopolymer resin formulation itself must be meticulously designed, selecting appropriate reactive monomers and precisely controlling photoinitiator concentrations to achieve optimal performance.

In particular, DLP technology has been widely adopted for the additive manufacturing of AlN ceramics due to its high precision, speed, cost efficiency, and compatibility with high-solid-loading ceramic suspensions. Recent research has shown several advancements. Rauchenecker et al. [24] conducted a comparative study between DLP-fabricated components and those produced via cold isostatic pressing, demonstrating that optimized DLP parameters can yield microstructures and thermo-mechanical properties on par with conventional techniques. Further studies by Lin et al. [25] explored the effects of various coupling agents on the rheological behavior, suspension stability, and curing characteristics of surface-modified AlN suspensions. Subsequent work by Lin et al. [26] revealed that fatty acid and oleic-acid-modified AlN powders exhibit improved wettability with the resin matrix, thereby enhancing green body uniformity. After sintering, the DLP-fabricated parts exhibited increased densification, higher flexural strength, and improved hardness. Duan et al. [27] investigated the influence of sintering temperature on the resulting microstructure, thermal conductivity, and mechanical performance of DLP-printed AlN ceramics. Sheng et al. [28] employed powder surface coating techniques to optimize both the rheology and curing kinetics of AlN suspensions, further contributing to microstructural uniformity and performance in the final ceramic. Additionally, Lin et al. [29] investigated the impact of the powder particle size on suspension rheology, curing depth, and sintering response, revealing a strong correlation with the thermal conductivity and flexural strength of the sintered AlN ceramics.

In these studies, it mainly focuses on the research of AlN ceramic powders, powder modifications, and sintering processes. However, for the key components in the AlN ceramic slurry, the photopolymer resin, which has a significant impact on the rheological properties of the slurry and printing accuracy, has received relatively little attention.

The monomers are the most critical component of the photopolymer resin. Based on the number of reactive functional groups per molecule, monomers can be classified into monofunctional, bifunctional, or multifunctional types. In photopolymer resin systems, monofunctional monomers demonstrate relatively low viscosity, significantly enhancing the resin’s rheological properties. However, they exhibit slow curing rates and yield cured materials with poor mechanical strength. In contrast, multifunctional monomers not only accelerate curing but also promote the formation of a densely cross-linked network structure, resulting in printed parts with superior mechanical performance. However, their high viscosity and significant shrinkage can adversely affect curing accuracy. The properties of bifunctional monomers are intermediate between those of monofunctional monomers and multifunctional monomers, typically serving to balance the characteristics of both [30].

The properties of monomers vary significantly depending on the type of functional groups, even when they contain the same number of functional groups. Therefore, considering the characteristics of different quantities of functional groups and the properties of each functional group, the research on multicomponent photopolymer resin systems has great significance for DLP 3D printing technology.

This study systematically compared a series of photosensitive resins incorporating monofunctional, bifunctional, and multifunctional monomers. Through a comprehensive characterization of resins and printed components, the optimal monomer composition and mixing ratio for enhanced performance were identified. Additionally, the solid loading of the prepared AlN ceramic slurry was optimized to identify the most suitable level for printing. Finally, using the formulated AlN ceramic slurry, through printing, debinding, and sintering, dense AlN ceramic components were successfully fabricated.

## 2. Materials and Methods

### 2.1. Materials

In this study, aluminum nitride powder (D50 = 1 µm, Shanghai ST-Nano Science & Technology Co., Ltd., Shanghai, China) was used, and yttria powder (D50 = 1 µm, Shanghai ST-Nano Science & Technology Co., Ltd., Shanghai, China) served as a sintering additive. The photoinitiator used was 2,4,6-trimethyl benzoyl diphenyl phosphine oxide (TPO, Shanghai Guangyi Chemical Co., Ltd., Shanghai, China), while KOS110 (Dongguan Haoyouduo New Materials Co., Ltd., Dongguan, China) was used as a dispersant. The photosensitive resin monomers used in this study are listed in Table 1.

### 2.2. Preparation and Characterization of Photopolymer Resin

The photopolymer resin was prepared by mixing different resin monomers using a magnetic stirrer (RCT-Basic, IKA-Werke GmbH & Co. KG, Staufen im Breisgau, Germany) at 500 r/min for 60 min; TOP was added with 3 wt% of resins as the photoinitiator.

After mixing, the viscosity of the resin was measured using a rotary viscometer (NDJ-8Spro, Shanghai Xiniulab Instruments Co., Ltd., Shanghai, China). The mixed resin was printed for test specimens, using a DLP resin printer (BLD-50-C1, Qingdao Breuck 3D Additive Manufacturing Co., Ltd., Qingdao, China). The dimensions of the printed specimens were 80 × 10 × 4 mm for bending tests and 75 × 10 × 3 mm for tensile tests. The lengths of the printed bending test specimens were measured using a vernier caliper (CD-10AX, Mitutoyo Corporation, Kawasaki, Japan), to evaluate the printing accuracy. The specimens were tested for tensile and bending strength using a digital electronic universal testing machine (WH-70, Ningbo Weiheng Testing Instruments Co., Ltd., Ningbo, China). The testing procedures followed the ISO 178:2001 [31] and ISO 527:2012 [32] standards; the details of printing and testing processes of tensile and bending strength were described in a previous study [33].

### 2.3. Preparation of AlN Ceramic Slurry

**Figure 1:**
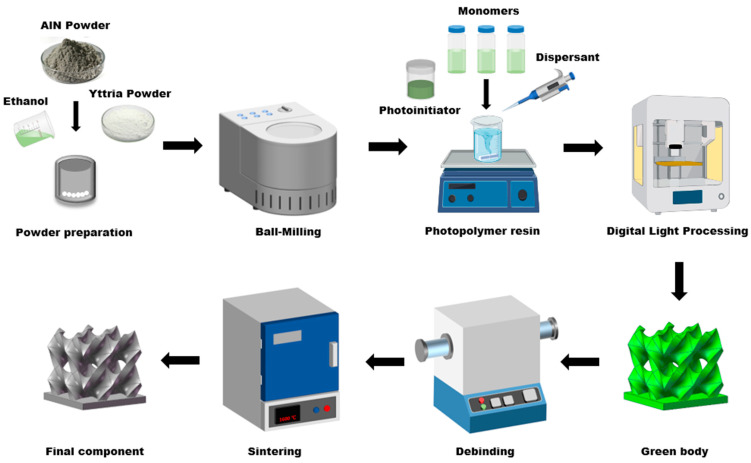
Three-dimensional printing AlN ceramic fabrication process.

The preparation process of the AlN slurry is shown in Figure 1. Initially, the AlN and yttria powders with a mass ratio of 95:5 were first dispersed in ethanol and subjected to planetary ball milling (BQM-1L, Changsha Miqi Instruments & Equipment Co., Ltd., Changsha, China) at 300 rpm for 8 h to effectively break down agglomerates. After milling, the ethanol was evaporated by drying the suspension at 60 °C. The dried powder was then sieved through a 100-mesh screen to ensure uniform particle size distribution. Next, a predetermined amount of the prepared powder was incorporated into the mixed photopolymer resin, dispersant KOS110 with 3 wt% of powder was added to the suspension, and the mixture was homogenized via ball milling for 10 min to obtain a uniform slurry.

### 2.4. AlN Ceramic Sample Fabrication and Characterization

AlN ceramic green bodies were printed using a DLP ceramic printer (BLD-25-C1, Qingdao Breuck 3D Additive Manufacturing Co., Ltd., Qingdao, China). The print thickness was selected as 30 µm, with the exposure power between 5 and 9 mW/cm^2^, and an exposure time of 1–30 s. After printing, the green body was tested via thermogravimetric analysis (TGA; STA8000 Frontier, PerkinElmer Corporation, Waltham, MA, USA), with a heating rate of 5 °C/min. Since TGA curves accurately characterize the mass loss stages of organic components at various temperatures, they served as the scientific basis for determining critical debinding parameters, including the heating rate, target temperature, and holding time, to achieve gradual and controlled organic removal. AlN green bodies were debound at 550 °C in a tube furnace (OTF-1200X, Hefei Kejing Materials Technology Co., Ltd., Hefei, China) in vacuum condition for 3 h and then sintered in an atmosphere furnace (ZT-18-22, Shanghai Chenhua Technology Co., Ltd., Shanghai, China) under a nitrogen atmosphere with a heating rate of 5 °C/min to 1800 °C for 4 h. The microstructure of the sintered sample was characterized using a scanning electron microscope (SEM, Regulus8100, HITACHI, Tokyo, Japan).

## 3. Results and Discussion

### 3.1. Formula Selection of Photopolymer Resin

In this study, we selected each of three monomers from monofunctional monomers, bifunctional monomers, and multifunctional monomers, as listed in Table 2, and composed the photopolymer resin formula used in the experiment. Therefore, there were a total of 27 different formula combinations consisting of various monomers in this experiment.

For a given formulation composition, three distinct monomers (i.e., three factors) were included. Due to experimental cost constraints, eight levels were assigned to each combination, resulting in eight distinct mixing ratios per formulation. Therefore, to ensure that the selected formulations are distributed as uniformly as possible across the experimental range, a uniform design table ${UM}_{8}^{*}(8^{3})$ [34] was employed to optimize the photopolymer resin formulations. The calculation process is as follows:

(1) $$C_{ji}=\frac{2q_{ji}-1}{2n},\;\;j=1,\;2,\;\ldots ,\;m-1,\;\;i=1,2,\ldots ,n,$$

(2) $$\left\{\begin{array}{c} x_{ji}=\left(1-C_{ji}^{\frac{1}{m-j}}\right)\prod_{k=1}^{j-1}C_{ki}^{\frac{1}{m-k}}\\ x_{mi}=\prod_{k=1}^{m-1}C_{ki}^{\frac{1}{m-k}} \end{array}\right.,$$

**Figure 2:**
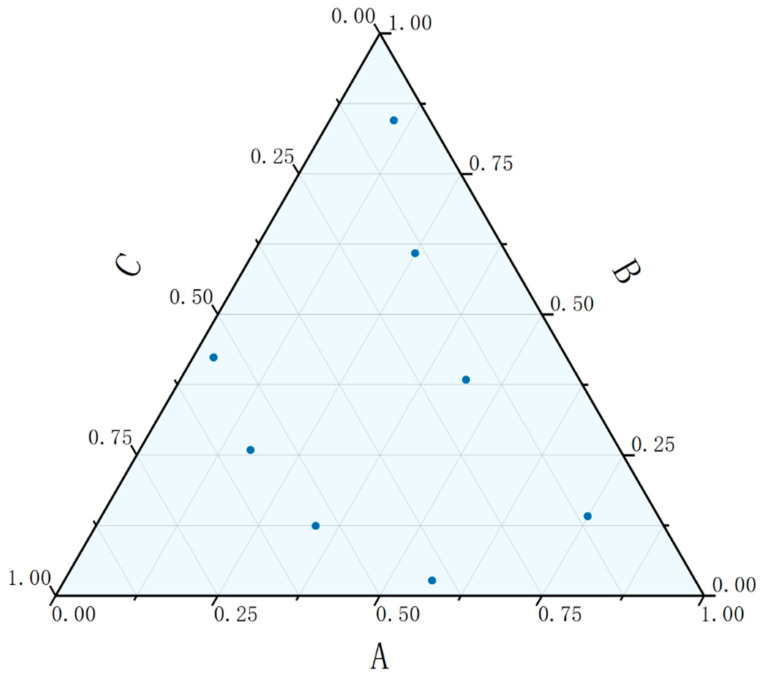
Distribution map of experimental points. A: Monofunctional; B: Bifunctional; C: Multifunctional.

Here, “m” represents the number of factors, and “n” is the number of experimental levels; $q_{ji}$ denotes the value in the “i”-th row and “j”-th column of the uniform design table with equal levels, where “i” ranges from 1 to 8 and “j” from 1 to 2. After calculation, the proportional composition of monofunctional monomers, bifunctional monomers, and multifunctional monomers is shown in Table 3, and its distribution map of experimental points is illustrated in Figure 2.

For example, ACMO, HDDA, and TMP3EOTA were selected as monofunctional monomers, bifunctional monomers, and multifunctional monomers respectively, and the proportion of their formulations was set at 75%, 14.1%, and 10.9% respectively. According to Table 2 and Table 3, it could be named as A1B1C1-1, and in the subsequent discussion, we simplified it to 111-1. Therefore, all the formulation proportions can be named as 111-1 to 333-8, totaling 216 groups, and the detailed compositions and proportions are shown in Table A1 of Appendix A.

### 3.2. Characterization of Photopolymer Resin

#### 3.2.1. Rheological Properties of Photopolymer Resin

**Figure 3:**
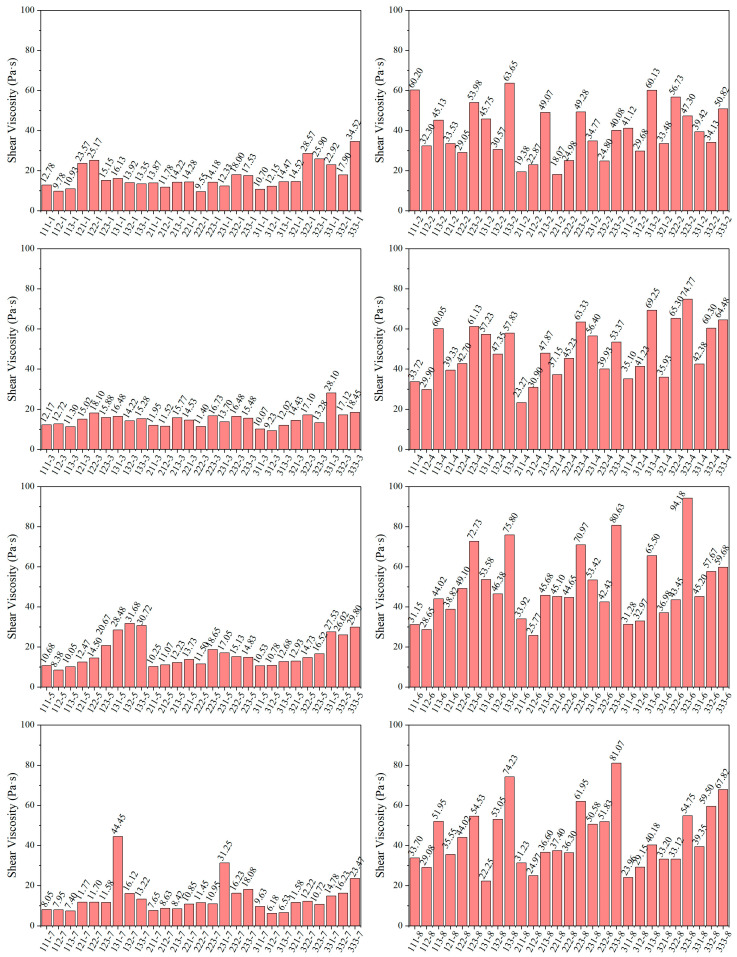
Shear viscosity of all resin groups.

The shear viscosity of 216 groups of photopolymer resin formulations was tested. According to the viscosity measurements, all tested resins exhibit Newtonian fluid behavior, maintaining constant viscosity under varying shear rates. The shear stress increases proportionally with the shear rate, and this difference becomes more pronounced at higher shear rates. Therefore, a shear rate of 12 s^−1^, which is relatively high within the equipment’s measurable range, was selected to compare the viscosities of the different photopolymer resins, as shown in Figure 3. The shear viscosity of group 312-7 was the lowest at only 6.18 Pa·s. Additionally, groups 313-7, 113-7, 211-7, and 112-7 also exhibited relatively low viscosities, all significantly below 7 Pa·s. On the contrary, the shear viscosity of group 323-6 was the highest, at 94.18 Pa·s, and the viscosities of groups 233-8 and 233-6 also exceeded 80 Pa·s.

From the overall results, it can be seen that the formulations with ratio groups of 2, 4, 6, and 8 have higher viscosities. This is because the proportion of multifunctional monomers in these groups is relatively high. Overall, the photopolymer resins in all groups demonstrated good flow properties, meeting the requirements for subsequent printing without adversely affecting its performance.

#### 3.2.2. Printing Precision

**Figure 4:**
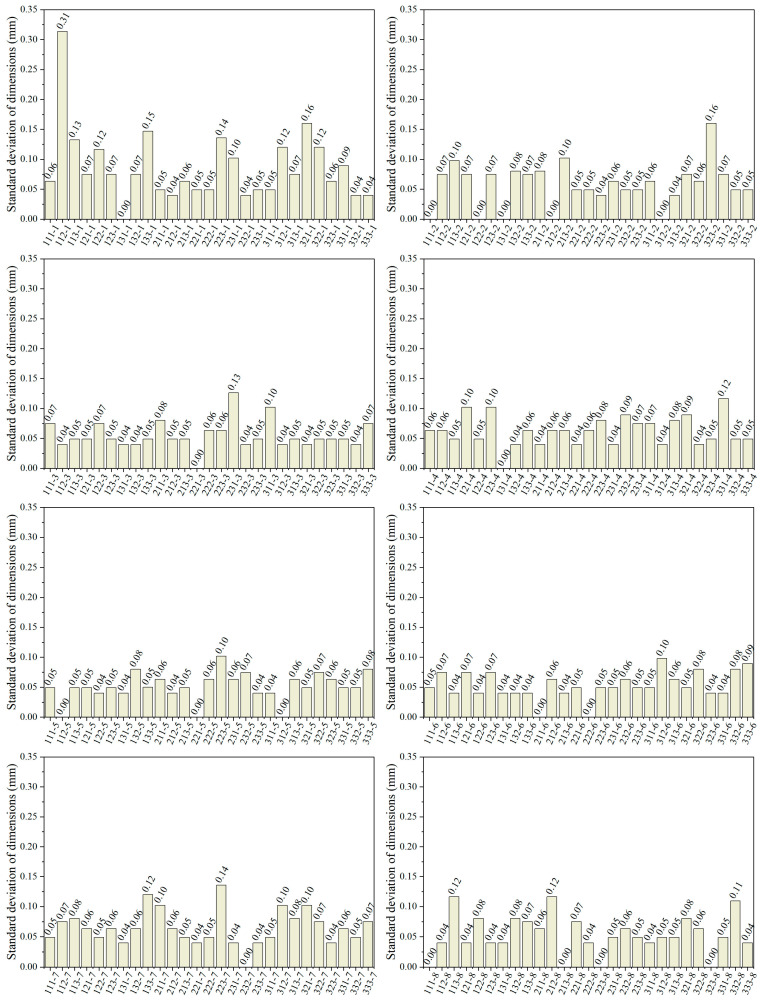
Standard dimension deviation of all resin groups.

The printing precision of the photopolymer resin was quantitatively assessed by determining the standard deviation [35], based on dimensional measurements of the fabricated components. The standard deviation of the dimensions can reflect the dispersion of the resin’s printed dimensions. The lower the standard deviation, the lower the dispersion of the data and the higher the photopolymerization precision. As shown in Figure 4, the standard deviation of 112-1 is the greatest, at 0.31 mm. Group 131-1 and the other 17 groups did not detect any deviations, demonstrating extremely high printing precision.

The overall results indicate no significant variation in standard deviation, suggesting that the photopolymer resin formulation ratio and viscosity exhibit minimal influence on printing precision.

#### 3.2.3. Tensile Strength

**Figure 5:**
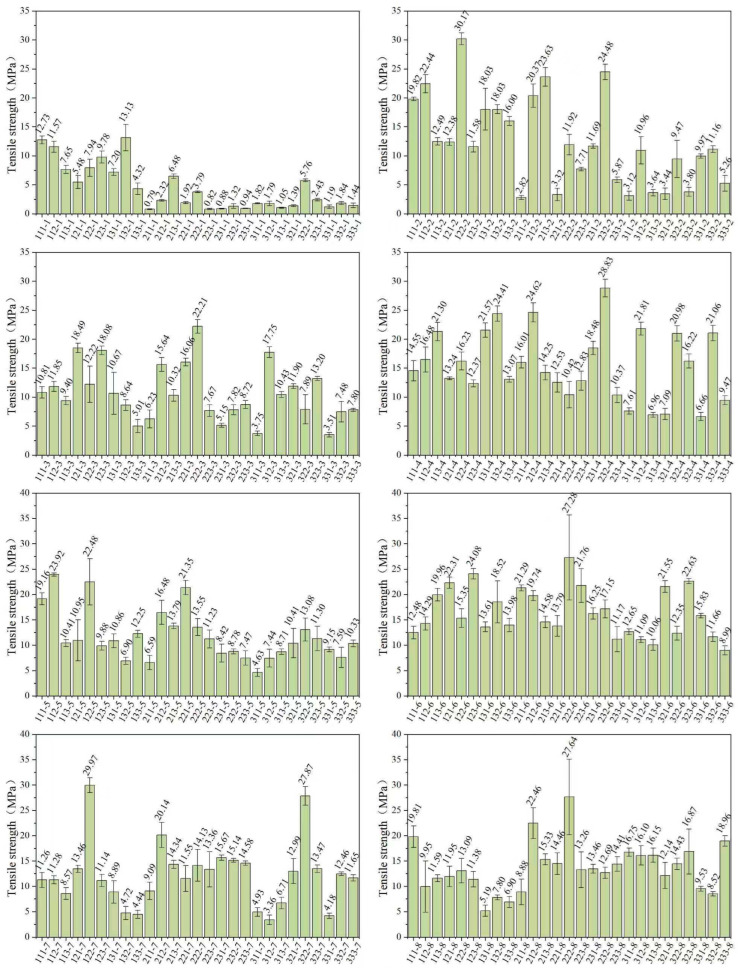
Tensile strength of all resin groups.

The tensile strength properties of the printed specimens are illustrated in Figure 5; the tensile strength of group 122-2 was the highest, at 30.17 MPa, and the tensile strengths of groups 122-7 and 232-4 also exceeded 28 MPa, but in contrast, the tensile strength of group 211-1 was only 0.79 MPa; groups 223-1, 231-1, and 233-1 also exhibited relatively low tensile strengths, all below 1 MPa.

The experimental results demonstrate that specimens printed with photopolymer resins from ratio groups 2, 4, 6, and 8 exhibit generally higher tensile strength compared to the other groups. This enhancement can be attributed to the higher content of multifunctional monomers in these resin groups. These findings corroborate that the cross-linked network structures formed during the photopolymerization of multifunctional monomers contribute to improved mechanical strength.

Furthermore, since the proportions of monofunctional monomers in the groups 1 and 2 are relatively high, the influence of the monofunctional monomers can be clearly compared. From the test results, it can be observed that the photopolymer containing the monomer ACMO has a significantly higher tensile strength compared to the other two monofunctional monomers. It is indicated that ACMO has a positive impact on promoting the cross-linking process.

#### 3.2.4. Bending Strength

**Figure 6:**
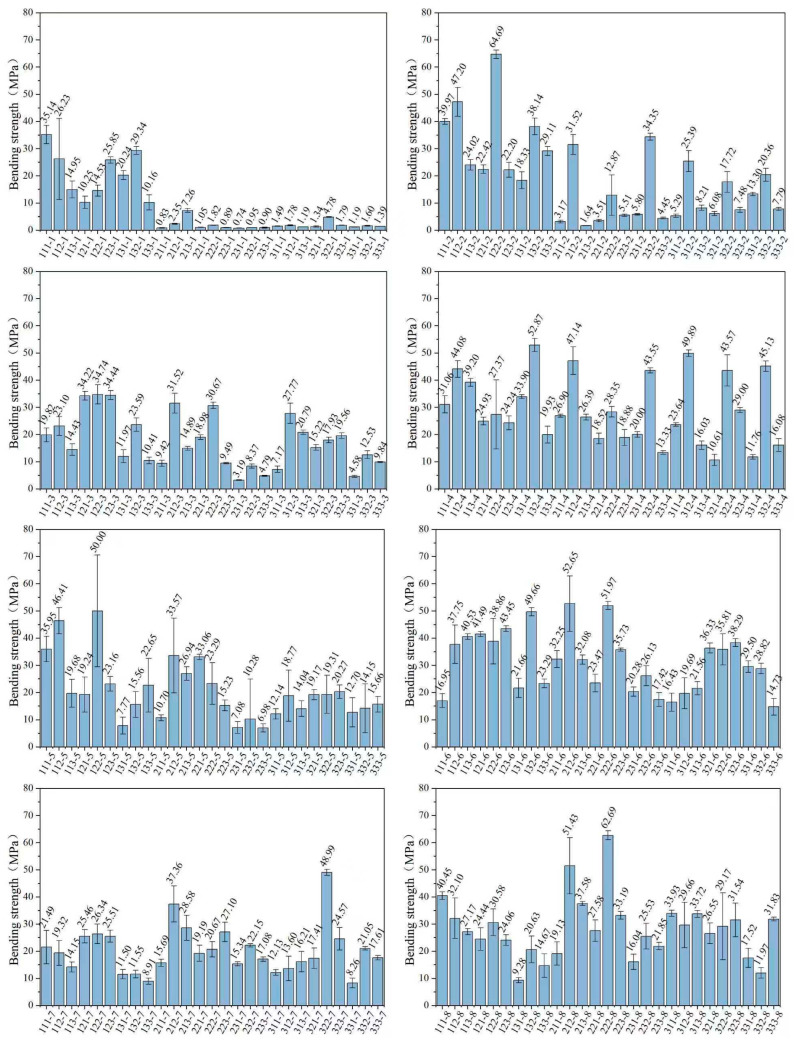
Bending strength of all resin groups.

Figure 6 presents the bending strength characteristics of the printed specimens. Among the tested groups, group 122-2 demonstrated the highest bending strength at 64.69 MPa, while group 222-8 also showed considerable strength exceeding 60 MPa. In marked contrast, group 231-1 exhibited significantly lower mechanical performance with a bending strength of merely 0.74 MPa. Similarly, groups 211-1, 223-1, 233-1, and 232-1 all displayed substantially reduced bending strengths, with values consistently below 1 MPa.

The overall bending strength results align closely with the tensile strength data, with photopolymer resins from ratio groups 2, 4, 6, and 8 consistently demonstrating superior bending performance compared to the other groups. The reason is also the same as that described in Section 3.2.3.

Furthermore, from the bending test results, it can also be observed that the bending strength of the photopolymer resin containing the monomer ACMO is significantly higher than that of the other two monofunctional monomers. This is consistent with the results of the tensile strength test.

#### 3.2.5. Comprehensive Assessment

In order to determine the optimal three-component photopolymer resin formula, the test data on the viscosity, printing precision, tensile strength, and bending strength of the photopolymer resin were summarized, and a comprehensive evaluation was conducted.

(3) $$V_{i}=\frac{U_{i}-U_{min}}{U_{max}-U_{min}},\;i=1,2,3,4,$$

(4) $$V_{i}=\frac{U_{max}-U_{i}}{U_{max}-U_{min}},\;i=1,2,3,4,$$

(5) $$W=\sum_{i=1}^{4}V_{i}x_{i},x_{1}=x_{2}=x_{3}=x_{4}=0.25$$

To calculate the overall score, the scores for each item are determined using Equations (3) and (4), where *i* represents the four criteria of viscosity, printing precision, tensile strength, and bending strength. Treating the four criteria as equally important, its weights *x_i_* (i = 1, 2, 3, 4) are all set to 0.25, and Equation (5) was used to calculate the composite scores of all formulation groups.

The comprehensive evaluation approach enables the integration of multiple assessment metrics, effectively mitigating the limitations associated with single-parameter optimization. This methodology facilitates a direct comparison of different formulations through computed composite scores or ranked evaluations, thereby supporting more informed decision-making processes. However, suboptimal weight assignment in the evaluation framework may inadvertently diminish the relative importance of critical performance parameters. Consequently, while the present study employs an equal-weight allocation strategy, future research could explore more sophisticated weighting schemes to enhance the evaluation system’s precision and applicability.

**Figure 7:**
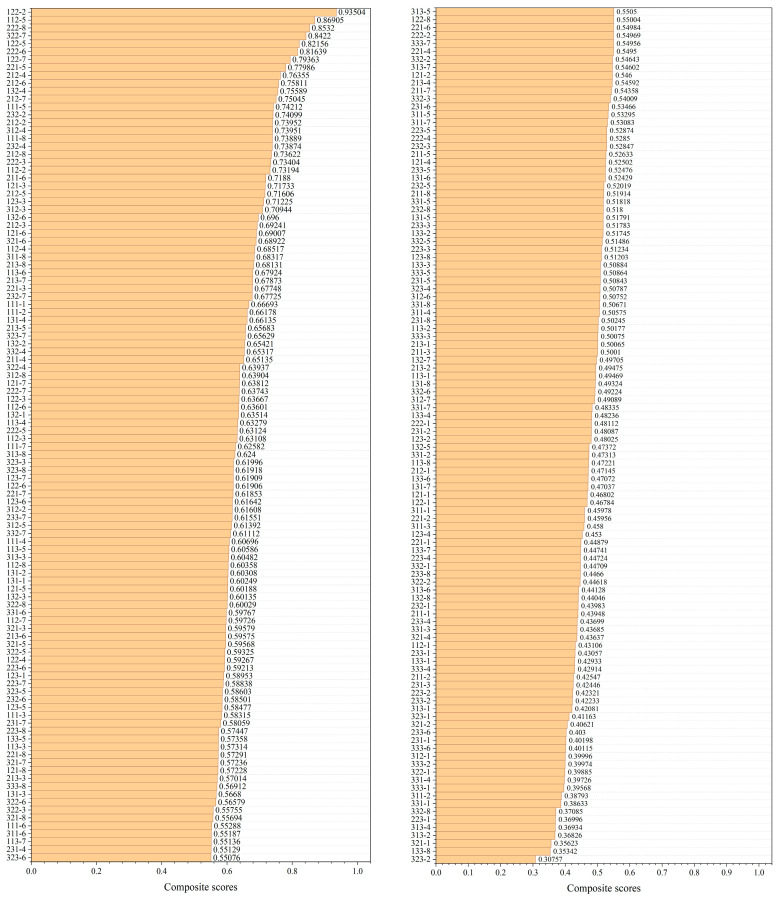
Composite scores of all resin groups.

As can be seen from Figure 7, the group 122-2 received the highest score of 0.93504, which was also the only one exceeding 0.9, the groups 112-5, 222-8, 322-7, 122-5, and 222-6 also achieved relatively high scores, all exceeding 0.8. The group 323-2, which received the lowest score, only achieved 0.30757, which is only one-third of the highest score. Based on the comprehensive evaluation scores, we selected the photopolymer resin formula of group 122-2 for use in the subsequent preparation of the AlN ceramic slurry.

### 3.3. Rheological Properties of AlN Slurry

**Figure 8:**
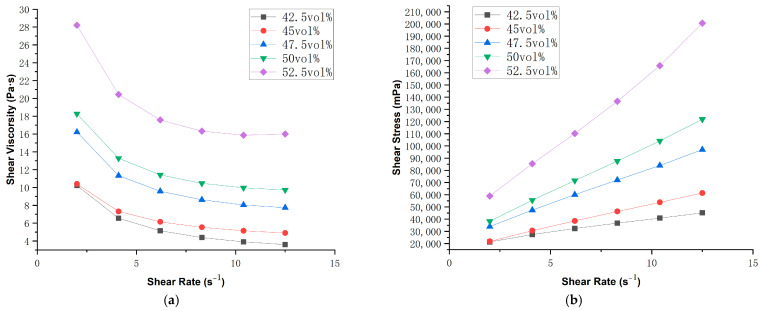
(**a**) Relationship between shear viscosity and shear rate of AlN ceramic slurry with different solid loadings; (**b**) relationship between shear stress and shear rate of AlN ceramic slurry with different solid loadings.

Under identical photosensitive resin formulations, the ceramic powder content in the ceramic slurry, i.e., solid loading plays a critical role in determining the rheological properties of the slurry. Systematic rheological characterization of the AlN ceramic suspension revealed the optimal solid loading range, as illustrated in Figure 8a. The results demonstrate a pronounced viscosity increase with higher solid loading, exhibiting a sharp viscosity surge at 52.5 vol% loading. Maintaining appropriate fluidity is paramount for successful printing, the viscosity should normally be less than 10 Pa·s at a 10 s^−1^ shear rate [36]. Measurements showed that the 52.5 vol% solid loading slurry reached 20 Pa·s viscosity at 10 s^−1^, exceeding printable limits. As presented in Figure 8b, the shear stress also increases with the increase in solid loading. The shear stresses at a typical shear rate of 10 s^−1^ were 40,889, 53,782, 83,898, 104,127, and 165,768 mPa for the slurries with solid loading of 42.5 vol%, 45 vol%, 47.5 vol%, 50 vol%, and 52 vol%, respectively. The shear stress exhibits a progressive increase with higher solid loading. However, beyond 50 vol%, this increase becomes substantially more dramatic. Consequently, the optimal solid loading for the AlN ceramic slurry was established at 50 vol% to ensure reliable printability.

In the current research on the 3D printing of AlN, the solid loading typically ranges from 43 vol% to 55 vol% [25,26,27,28,29]. The 50 vol% solid loading slurry prepared in this study therefore represents a relatively reasonable solid loading. Moreover, this solid loading level significantly exceeds the <45 vol% threshold typical of conventional tape casting processes [37], while remaining comparable to the 45 vol%–60 vol% range employed in injection molding techniques [38].

### 3.4. TG-DTG Analysis

**Figure 9:**
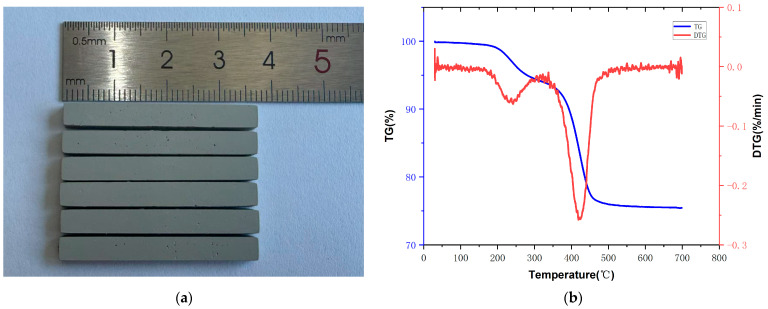
(**a**) Printed AlN ceramic green body; (**b**) TG-DTG curve of printed AlN ceramic green body.

The printed components with resin group 112-2 (ACMO 56.7 wt%, DEGDA 2.7 wt% and TMPTA 40.6 wt%) are presented in Figure 9a, and the TG-DTG curve in Figure 9b shows the thermogravimetric analysis results with this component. The TG-DTG analysis reveals two distinct decomposition peaks, a primary peak occurring at 200–300 °C followed by a more intense secondary peak in the 350–500 °C range. Complete decomposition was essentially attained at 550 °C, as indicated by the subsequent stabilization of the green body mass. Based on the above results, a debinding curve was set up. Under a vacuum environment, the initial heating rate was set at 1 °C/min from room temperature to 200 °C, followed by a reduced rate of 0.2 °C/min through the critical decomposition range 200 °C to 550 °C, and the green body was held at 550 °C for 3 h to ensure that the decomposition products of the polymerized macromolecules could escape and there were no defects inside the green body.

### 3.5. Properties of AlN Ceramics

**Figure 10:**
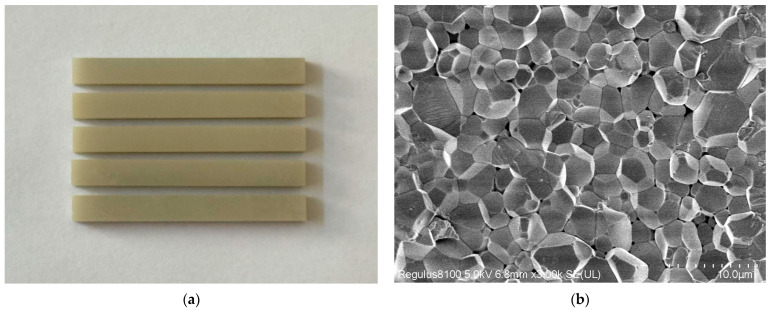
(**a**) Sintered AlN ceramic components; (**b**) microstructure of sintered AlN ceramic.

The sintered components, fabricated from resin group 112-2 with a composition of 56.7 wt% ACMO, 2.7 wt% DEGDA, and 40.6 wt% TMPTA, are displayed in Figure 10a. The microstructure of the sintered components was analyzed, as illustrated in Figure 10b. The SEM micrographs reveal a densely packed polycrystalline morphology in the sintered specimens, indicating that it already possesses excellent mechanical properties. With residual micro-scale pores implying suboptimal sintering conditions, this necessitates refinement of the thermal processing parameters to achieve full densification.

## 4. Conclusions

In this study, the photopolymer resin system used for DLP AlN ceramic additive manufacturing was systematically studied. By selecting a variety of monofunctional, bifunctional, and multifunctional monomers, a large number of photopolymer resins with different combinations and proportions were prepared, and an optimal combination of photopolymer resin monomers and their formulation ratios were determined through a comprehensive evaluation. The results showed that when monomers ACMO, DEGDA, and TMPTA are mixed in a ratio of 56.7 wt%, 2.7 wt%, and 40.6 wt%, the comprehensive performance of the photopolymer resin was the best. Through the study of the solid loading of the AlN ceramic slurry, the optimal solid loading suitable for printing was determined to be 50 vol%. After printing, debinding, and sintering, finally, relatively dense AlN ceramic samples were prepared.

This study once again demonstrates that the DLP additive manufacturing process can produce AlN ceramic components, which also creates the possibility for the manufacture of high-performance AlN ceramic devices with complex structures.

## Figures and Tables

**Table 1:** Monomers used in the experiment.

Monomer Name	Abbreviation	Number of Functional Groups	Source
Acryloyl morpholine	ACMO	1	Shanghai Guangyi Chemical Co., Ltd., Shanghai, China
2-Phenoxyethyl acrylate	PHEA	1	Shanghai Guangyi Chemical Co., Ltd., Shanghai, China
5-ethyl-1,3-dioxan-5-yl methyl acrylate	CTFA	1	Shanghai Guangyi Chemical Co., Ltd., Shanghai, China
1,6-Hexanediol diacrylate	HDDA	2	Shanghai Guangyi Chemical Co., Ltd., Shanghai, China
Diethylene glycol diacrylate	DEGDA	2	Guangzhou Goliang Technology Co., Ltd., Guangzhou, China
Tripropylene glycol diacrylate	TPGDA	2	Shanghai Guangyi Chemical Co., Ltd., Shanghai, China
Ethoxylated trimethylolpropane triacrylate	TMP3EOTA	3	Shanghai Guangyi Chemical Co., Ltd., Shanghai, China
Trimethylolpropane triacrylate	TMPTA	3	Shanghai Guangyi Chemical Co., Ltd., Shanghai, China
Ethoxylated pentaerythritol tetraacrylate	PPTTA	4	Shanghai Guangyi Chemical Co., Ltd., Shanghai, China

**Table 2:** Monomers of resin.

No.	A (Monofunctional)	B (Bifunctional)	C (Multifunctional)
1	ACMO	HDDA	TMP3EOTA
2	PHEA	DEGDA	TMPTA
3	CTFA	TPGDA	PPTTA

**Table 3:** Proportional composition of resin.

No.	A (wt%)	B (wt%)	C (wt%)
1	75.0	14.1	10.9
2	56.7	2.7	40.6
3	44.1	38.4	17.5
4	33.9	12.4	53.7
5	25.0	60.9	14.1
6	17.1	25.9	57.0
7	9.9	84.5	5.6
8	3.2	42.4	54.4

## Data Availability

The original contributions presented in this study are included in the article. Further inquiries can be directed to the corresponding authors.

## Appendix A

**Table A1:** Detailed composition of all resin groups.

Resin Group	Monomer A (wt%)	Monomer B (wt%)	Monomer C (wt%)
111-1	ACMO (75.0 wt%)	HDDA (14.1 wt%)	TMP3EOTA (10.9 wt%)
112-1	ACMO (75.0 wt%)	HDDA (14.1 wt%)	TMPTA (10.9 wt%)
113-1	ACMO (75.0 wt%)	HDDA (14.1 wt%)	PPTTA (10.9 wt%)
121-1	ACMO (75.0 wt%)	DEGDA (14.1 wt%)	TMP3EOTA (10.9 wt%)
122-1	ACMO (75.0 wt%)	DEGDA (14.1 wt%)	TMPTA (10.9 wt%)
123-1	ACMO (75.0 wt%)	DEGDA (14.1 wt%)	PPTTA (10.9 wt%)
131-1	ACMO (75.0 wt%)	TPGDA (14.1 wt%)	TMP3EOTA (10.9 wt%)
132-1	ACMO (75.0 wt%)	TPGDA (14.1 wt%)	TMPTA (10.9 wt%)
133-1	ACMO (75.0 wt%)	TPGDA (14.1 wt%)	PPTTA (10.9 wt%)
211-1	PHEA (75.0 wt%)	HDDA (14.1 wt%)	TMP3EOTA (10.9 wt%)
212-1	PHEA (75.0 wt%)	HDDA (14.1 wt%)	TMPTA (10.9 wt%)
213-1	PHEA (75.0 wt%)	HDDA (14.1 wt%)	PPTTA (10.9 wt%)
221-1	PHEA (75.0 wt%)	DEGDA (14.1 wt%)	TMP3EOTA (10.9 wt%)
222-1	PHEA (75.0 wt%)	DEGDA (14.1 wt%)	TMPTA (10.9 wt%)
223-1	PHEA (75.0 wt%)	DEGDA (14.1 wt%)	PPTTA (10.9 wt%)
231-1	PHEA (75.0 wt%)	TPGDA (14.1 wt%)	TMP3EOTA (10.9 wt%)
232-1	PHEA (75.0 wt%)	TPGDA (14.1 wt%)	TMPTA (10.9 wt%)
233-1	PHEA (75.0 wt%)	TPGDA (14.1 wt%)	PPTTA (10.9 wt%)
311-1	CTFA (75.0 wt%)	HDDA (14.1 wt%)	TMP3EOTA (10.9 wt%)
312-1	CTFA (75.0 wt%)	HDDA (14.1 wt%)	TMPTA (10.9 wt%)
313-1	CTFA (75.0 wt%)	HDDA (14.1 wt%)	PPTTA (10.9 wt%)
321-1	CTFA (75.0 wt%)	DEGDA (14.1 wt%)	TMP3EOTA (10.9 wt%)
322-1	CTFA (75.0 wt%)	DEGDA (14.1 wt%)	TMPTA (10.9 wt%)
323-1	CTFA (75.0 wt%)	DEGDA (14.1 wt%)	PPTTA (10.9 wt%)
331-1	CTFA (75.0 wt%)	TPGDA (14.1 wt%)	TMP3EOTA (10.9 wt%)
332-1	CTFA (75.0 wt%)	TPGDA (14.1 wt%)	TMPTA (10.9 wt%)
333-1	CTFA (75.0 wt%)	TPGDA (14.1 wt%)	PPTTA (10.9 wt%)
111-2	ACMO (57.6 wt%)	HDDA (2.7 wt%)	TMP3EOTA (40.6 wt%)
112-2	ACMO (57.6 wt%)	HDDA (2.7 wt%)	TMPTA (40.6 wt%)
113-2	ACMO (57.6 wt%)	HDDA (2.7 wt%)	PPTTA (40.6 wt%)
121-2	ACMO (57.6 wt%)	DEGDA (2.7 wt%)	TMP3EOTA (40.6 wt%)
122-2	ACMO (57.6 wt%)	DEGDA (2.7 wt%)	TMPTA (40.6 wt%)
123-2	ACMO (57.6 wt%)	DEGDA (2.7 wt%)	PPTTA (40.6 wt%)
131-2	ACMO (57.6 wt%)	TPGDA (2.7 wt%)	TMP3EOTA (40.6 wt%)
132-2	ACMO (57.6 wt%)	TPGDA (2.7 wt%)	TMPTA (40.6 wt%)
133-2	ACMO (57.6 wt%)	TPGDA (2.7 wt%)	PPTTA (40.6 wt%)
211-2	PHEA (57.6 wt%)	HDDA (2.7 wt%)	TMP3EOTA (40.6 wt%)
212-2	PHEA (57.6 wt%)	HDDA (2.7 wt%)	TMPTA (40.6 wt%)
213-2	PHEA (57.6 wt%)	HDDA (2.7 wt%)	PPTTA (40.6 wt%)
221-2	PHEA (57.6 wt%)	DEGDA (2.7 wt%)	TMP3EOTA (40.6 wt%)
222-2	PHEA (57.6 wt%)	DEGDA (2.7 wt%)	TMPTA (40.6 wt%)
223-2	PHEA (57.6 wt%)	DEGDA (2.7 wt%)	PPTTA (40.6 wt%)
231-2	PHEA (57.6 wt%)	TPGDA (2.7 wt%)	TMP3EOTA (40.6 wt%)
232-2	PHEA (57.6 wt%)	TPGDA (2.7 wt%)	TMPTA (40.6 wt%)
233-2	PHEA (57.6 wt%)	TPGDA (2.7 wt%)	PPTTA (40.6 wt%)
311-2	CTFA (57.6 wt%)	HDDA (2.7 wt%)	TMP3EOTA (40.6 wt%)
312-2	CTFA (57.6 wt%)	HDDA (2.7 wt%)	TMPTA (40.6 wt%)
313-2	CTFA (57.6 wt%)	HDDA (2.7 wt%)	PPTTA (40.6 wt%)
321-2	CTFA (57.6 wt%)	DEGDA (2.7 wt%)	TMP3EOTA (40.6 wt%)
322-2	CTFA (57.6 wt%)	DEGDA (2.7 wt%)	TMPTA (40.6 wt%)
323-2	CTFA (57.6 wt%)	DEGDA (2.7 wt%)	PPTTA (40.6 wt%)
331-2	CTFA (57.6 wt%)	TPGDA (2.7 wt%)	TMP3EOTA (40.6 wt%)
332-2	CTFA (57.6 wt%)	TPGDA (2.7 wt%)	TMPTA (40.6 wt%)
333-2	CTFA (57.6 wt%)	TPGDA (2.7 wt%)	PPTTA (40.6 wt%)
111-3	ACMO (44.1 wt%)	HDDA (38.4 wt%)	TMP3EOTA (17.5 wt%)
112-3	ACMO (44.1 wt%)	HDDA (38.4 wt%)	TMPTA (17.5 wt%)
113-3	ACMO (44.1 wt%)	HDDA (38.4 wt%)	PPTTA (17.5 wt%)
121-3	ACMO (44.1 wt%)	DEGDA (38.4 wt%)	TMP3EOTA (17.5 wt%)
122-3	ACMO (44.1 wt%)	DEGDA (38.4 wt%)	TMPTA (17.5 wt%)
123-3	ACMO (44.1 wt%)	DEGDA (38.4 wt%)	PPTTA (17.5 wt%)
131-3	ACMO (44.1 wt%)	TPGDA (38.4 wt%)	TMP3EOTA (17.5 wt%)
132-3	ACMO (44.1 wt%)	TPGDA (38.4 wt%)	TMPTA (17.5 wt%)
133-3	ACMO (44.1 wt%)	TPGDA (38.4 wt%)	PPTTA (17.5 wt%)
211-3	PHEA (44.1 wt%)	HDDA (38.4 wt%)	TMP3EOTA (17.5 wt%)
212-3	PHEA (44.1 wt%)	HDDA (38.4 wt%)	TMPTA (17.5 wt%)
213-3	PHEA (44.1 wt%)	HDDA (38.4 wt%)	PPTTA (17.5 wt%)
221-3	PHEA (44.1 wt%)	DEGDA (38.4 wt%)	TMP3EOTA (17.5 wt%)
222-3	PHEA (44.1 wt%)	DEGDA (38.4 wt%)	TMPTA (17.5 wt%)
223-3	PHEA (44.1 wt%)	DEGDA (38.4 wt%)	PPTTA (17.5 wt%)
231-3	PHEA (44.1 wt%)	TPGDA (38.4 wt%)	TMP3EOTA (17.5 wt%)
232-3	PHEA (44.1 wt%)	TPGDA (38.4 wt%)	TMPTA (17.5 wt%)
233-3	PHEA (44.1 wt%)	TPGDA (38.4 wt%)	PPTTA (17.5 wt%)
311-3	CTFA (44.1 wt%)	HDDA (38.4 wt%)	TMP3EOTA (17.5 wt%)
312-3	CTFA (44.1 wt%)	HDDA (38.4 wt%)	TMPTA (17.5 wt%)
313-3	CTFA (44.1 wt%)	HDDA (38.4 wt%)	PPTTA (17.5 wt%)
321-3	CTFA (44.1 wt%)	DEGDA (38.4 wt%)	TMP3EOTA (17.5 wt%)
322-3	CTFA (44.1 wt%)	DEGDA (38.4 wt%)	TMPTA (17.5 wt%)
323-3	CTFA (44.1 wt%)	DEGDA (38.4 wt%)	PPTTA (17.5 wt%)
331-3	CTFA (44.1 wt%)	TPGDA (38.4 wt%)	TMP3EOTA (17.5 wt%)
332-3	CTFA (44.1 wt%)	TPGDA (38.4 wt%)	TMPTA (17.5 wt%)
333-3	CTFA (44.1 wt%)	TPGDA (38.4 wt%)	PPTTA (17.5 wt%)
111-4	ACMO (33.9 wt%)	HDDA (12.4 wt%)	TMP3EOTA (53.7 wt%)
112-4	ACMO (33.9 wt%)	HDDA (12.4 wt%)	TMPTA (53.7 wt%)
113-4	ACMO (33.9 wt%)	HDDA (12.4 wt%)	PPTTA (53.7 wt%)
121-4	ACMO (33.9 wt%)	DEGDA (12.4 wt%)	TMP3EOTA (53.7 wt%)
122-4	ACMO (33.9 wt%)	DEGDA (12.4 wt%)	TMPTA (53.7 wt%)
123-4	ACMO (33.9 wt%)	DEGDA (12.4 wt%)	PPTTA (53.7 wt%)
131-4	ACMO (33.9 wt%)	TPGDA (12.4 wt%)	TMP3EOTA (53.7 wt%)
132-4	ACMO (33.9 wt%)	TPGDA (12.4 wt%)	TMPTA (53.7 wt%)
133-4	ACMO (33.9 wt%)	TPGDA (12.4 wt%)	PPTTA (53.7 wt%)
211-4	PHEA (33.9 wt%)	HDDA (12.4 wt%)	TMP3EOTA (53.7 wt%)
212-4	PHEA (33.9 wt%)	HDDA (12.4 wt%)	TMPTA (53.7 wt%)
213-4	PHEA (33.9 wt%)	HDDA (12.4 wt%)	PPTTA (53.7 wt%)
221-4	PHEA (33.9 wt%)	DEGDA (12.4 wt%)	TMP3EOTA (53.7 wt%)
222-4	PHEA (33.9 wt%)	DEGDA (12.4 wt%)	TMPTA (53.7 wt%)
223-4	PHEA (33.9 wt%)	DEGDA (12.4 wt%)	PPTTA (53.7 wt%)
231-4	PHEA (33.9 wt%)	TPGDA (12.4 wt%)	TMP3EOTA (53.7 wt%)
232-4	PHEA (33.9 wt%)	TPGDA (12.4 wt%)	TMPTA (53.7 wt%)
233-4	PHEA (33.9 wt%)	TPGDA (12.4 wt%)	PPTTA (53.7 wt%)
311-4	CTFA (33.9 wt%)	HDDA (12.4 wt%)	TMP3EOTA (53.7 wt%)
312-4	CTFA (33.9 wt%)	HDDA (12.4 wt%)	TMPTA (53.7 wt%)
313-4	CTFA (33.9 wt%)	HDDA (12.4 wt%)	PPTTA (53.7 wt%)
321-4	CTFA (33.9 wt%)	DEGDA (12.4 wt%)	TMP3EOTA (53.7 wt%)
322-4	CTFA (33.9 wt%)	DEGDA (12.4 wt%)	TMPTA (53.7 wt%)
323-4	CTFA (33.9 wt%)	DEGDA (12.4 wt%)	PPTTA (53.7 wt%)
331-4	CTFA (33.9 wt%)	TPGDA (12.4 wt%)	TMP3EOTA (53.7 wt%)
332-4	CTFA (33.9 wt%)	TPGDA (12.4 wt%)	TMPTA (53.7 wt%)
333-4	CTFA (33.9 wt%)	TPGDA (12.4 wt%)	PPTTA (53.7 wt%)
111-5	ACMO (25.0 wt%)	HDDA (60.9 wt%)	TMP3EOTA (14.1 wt%)
112-5	ACMO (25.0 wt%)	HDDA (60.9 wt%)	TMPTA (14.1 wt%)
113-5	ACMO (25.0 wt%)	HDDA (60.9 wt%)	PPTTA (14.1 wt%)
121-5	ACMO (25.0 wt%)	DEGDA (60.9 wt%)	TMP3EOTA (14.1 wt%)
122-5	ACMO (25.0 wt%)	DEGDA (60.9 wt%)	TMPTA (14.1 wt%)
123-5	ACMO (25.0 wt%)	DEGDA (60.9 wt%)	PPTTA (14.1 wt%)
131-5	ACMO (25.0 wt%)	TPGDA (60.9 wt%)	TMP3EOTA (14.1 wt%)
132-5	ACMO (25.0 wt%)	TPGDA (60.9 wt%)	TMPTA (14.1 wt%)
133-5	ACMO (25.0 wt%)	TPGDA (60.9 wt%)	PPTTA (14.1 wt%)
211-5	PHEA (25.0 wt%)	HDDA (60.9 wt%)	TMP3EOTA (14.1 wt%)
212-5	PHEA (25.0 wt%)	HDDA (60.9 wt%)	TMPTA (14.1 wt%)
213-5	PHEA (25.0 wt%)	HDDA (60.9 wt%)	PPTTA (14.1 wt%)
221-5	PHEA (25.0 wt%)	DEGDA (60.9 wt%)	TMP3EOTA (14.1 wt%)
222-5	PHEA (25.0 wt%)	DEGDA (60.9 wt%)	TMPTA (14.1 wt%)
223-5	PHEA (25.0 wt%)	DEGDA (60.9 wt%)	PPTTA (14.1 wt%)
231-5	PHEA (25.0 wt%)	TPGDA (60.9 wt%)	TMP3EOTA (14.1 wt%)
232-5	PHEA (25.0 wt%)	TPGDA (60.9 wt%)	TMPTA (14.1 wt%)
233-5	PHEA (25.0 wt%)	TPGDA (60.9 wt%)	PPTTA (14.1 wt%)
311-5	CTFA (25.0 wt%)	HDDA (60.9 wt%)	TMP3EOTA (14.1 wt%)
312-5	CTFA (25.0 wt%)	HDDA (60.9 wt%)	TMPTA (14.1 wt%)
313-5	CTFA (25.0 wt%)	HDDA (60.9 wt%)	PPTTA (14.1 wt%)
321-5	CTFA (25.0 wt%)	DEGDA (60.9 wt%)	TMP3EOTA (14.1 wt%)
322-5	CTFA (25.0 wt%)	DEGDA (60.9 wt%)	TMPTA (14.1 wt%)
323-5	CTFA (25.0 wt%)	DEGDA (60.9 wt%)	PPTTA (14.1 wt%)
331-5	CTFA (25.0 wt%)	TPGDA (60.9 wt%)	TMP3EOTA (14.1 wt%)
332-5	CTFA (25.0 wt%)	TPGDA (60.9 wt%)	TMPTA (14.1 wt%)
333-5	CTFA (25.0 wt%)	TPGDA (60.9 wt%)	PPTTA (14.1 wt%)
111-6	ACMO (17.1 wt%)	HDDA (25.9 wt%)	TMP3EOTA (57.0 wt%)
112-6	ACMO (17.1 wt%)	HDDA (25.9 wt%)	TMPTA (57.0 wt%)
113-6	ACMO (17.1 wt%)	HDDA (25.9 wt%)	PPTTA (57.0 wt%)
121-6	ACMO (17.1 wt%)	DEGDA (25.9 wt%)	TMP3EOTA (57.0 wt%)
122-6	ACMO (17.1 wt%)	DEGDA (25.9 wt%)	TMPTA (57.0 wt%)
123-6	ACMO (17.1 wt%)	DEGDA (25.9 wt%)	PPTTA (57.0 wt%)
131-6	ACMO (17.1 wt%)	TPGDA (25.9 wt%)	TMP3EOTA (57.0 wt%)
132-6	ACMO (17.1 wt%)	TPGDA (25.9 wt%)	TMPTA (57.0 wt%)
133-6	ACMO (17.1 wt%)	TPGDA (25.9 wt%)	PPTTA (57.0 wt%)
211-6	PHEA (17.1 wt%)	HDDA (25.9 wt%)	TMP3EOTA (57.0 wt%)
212-6	PHEA (17.1 wt%)	HDDA (25.9 wt%)	TMPTA (57.0 wt%)
213-6	PHEA (17.1 wt%)	HDDA (25.9 wt%)	PPTTA (57.0 wt%)
221-6	PHEA (17.1 wt%)	DEGDA (25.9 wt%)	TMP3EOTA (57.0 wt%)
222-6	PHEA (17.1 wt%)	DEGDA (25.9 wt%)	TMPTA (57.0 wt%)
223-6	PHEA (17.1 wt%)	DEGDA (25.9 wt%)	PPTTA (57.0 wt%)
231-6	PHEA (17.1 wt%)	TPGDA (25.9 wt%)	TMP3EOTA (57.0 wt%)
232-6	PHEA (17.1 wt%)	TPGDA (25.9 wt%)	TMPTA (57.0 wt%)
233-6	PHEA (17.1 wt%)	TPGDA (25.9 wt%)	PPTTA (57.0 wt%)
311-6	CTFA (17.1 wt%)	HDDA (25.9 wt%)	TMP3EOTA (57.0 wt%)
312-6	CTFA (17.1 wt%)	HDDA (25.9 wt%)	TMPTA (57.0 wt%)
313-6	CTFA (17.1 wt%)	HDDA (25.9 wt%)	PPTTA (57.0 wt%)
321-6	CTFA (17.1 wt%)	DEGDA (25.9 wt%)	TMP3EOTA (57.0 wt%)
322-6	CTFA (17.1 wt%)	DEGDA (25.9 wt%)	TMPTA (57.0 wt%)
323-6	CTFA (17.1 wt%)	DEGDA (25.9 wt%)	PPTTA (57.0 wt%)
331-6	CTFA (17.1 wt%)	TPGDA (25.9 wt%)	TMP3EOTA (57.0 wt%)
332-6	CTFA (17.1 wt%)	TPGDA (25.9 wt%)	TMPTA (57.0 wt%)
333-6	CTFA (17.1 wt%)	TPGDA (25.9 wt%)	PPTTA (57.0 wt%)
111-7	ACMO (9.9 wt%)	HDDA (84.5 wt%)	TMP3EOTA (5.6 wt%)
112-7	ACMO (9.9 wt%)	HDDA (84.5 wt%)	TMPTA (5.6 wt%)
113-7	ACMO (9.9 wt%)	HDDA (84.5 wt%)	PPTTA (5.6 wt%)
121-7	ACMO (9.9 wt%)	DEGDA (84.5 wt%)	TMP3EOTA (5.6 wt%)
122-7	ACMO (9.9 wt%)	DEGDA (84.5 wt%)	TMPTA (5.6 wt%)
123-7	ACMO (9.9 wt%)	DEGDA (84.5 wt%)	PPTTA (5.6 wt%)
131-7	ACMO (9.9 wt%)	TPGDA (84.5 wt%)	TMP3EOTA (5.6 wt%)
132-7	ACMO (9.9 wt%)	TPGDA (84.5 wt%)	TMPTA (5.6 wt%)
133-7	ACMO (9.9 wt%)	TPGDA (84.5 wt%)	PPTTA (5.6 wt%)
211-7	PHEA (9.9 wt%)	HDDA (84.5 wt%)	TMP3EOTA (5.6 wt%)
212-7	PHEA (9.9 wt%)	HDDA (84.5 wt%)	TMPTA (5.6 wt%)
213-7	PHEA (9.9 wt%)	HDDA (84.5 wt%)	PPTTA (5.6 wt%)
221-7	PHEA (9.9 wt%)	DEGDA (84.5 wt%)	TMP3EOTA (5.6 wt%)
222-7	PHEA (9.9 wt%)	DEGDA (84.5 wt%)	TMPTA (5.6 wt%)
223-7	PHEA (9.9 wt%)	DEGDA (84.5 wt%)	PPTTA (5.6 wt%)
231-7	PHEA (9.9 wt%)	TPGDA (84.5 wt%)	TMP3EOTA (5.6 wt%)
232-7	PHEA (9.9 wt%)	TPGDA (84.5 wt%)	TMPTA (5.6 wt%)
233-7	PHEA (9.9 wt%)	TPGDA (84.5 wt%)	PPTTA (5.6 wt%)
311-7	CTFA (9.9 wt%)	HDDA (84.5 wt%)	TMP3EOTA (5.6 wt%)
312-7	CTFA (9.9 wt%)	HDDA (84.5 wt%)	TMPTA (5.6 wt%)
313-7	CTFA (9.9 wt%)	HDDA (84.5 wt%)	PPTTA (5.6 wt%)
321-7	CTFA (9.9 wt%)	DEGDA (84.5 wt%)	TMP3EOTA (5.6 wt%)
322-7	CTFA (9.9 wt%)	DEGDA (84.5 wt%)	TMPTA (5.6 wt%)
323-7	CTFA (9.9 wt%)	DEGDA (84.5 wt%)	PPTTA (5.6 wt%)
331-7	CTFA (9.9 wt%)	TPGDA (84.5 wt%)	TMP3EOTA (5.6 wt%)
332-7	CTFA (9.9 wt%)	TPGDA (84.5 wt%)	TMPTA (5.6 wt%)
333-7	CTFA (9.9 wt%)	TPGDA (84.5 wt%)	PPTTA (5.6 wt%)
111-8	ACMO (3.2 wt%)	HDDA (42.4 wt%)	TMP3EOTA (54.4 wt%)
112-8	ACMO (3.2 wt%)	HDDA (42.4 wt%)	TMPTA (54.4 wt%)
113-8	ACMO (3.2 wt%)	HDDA (42.4 wt%)	PPTTA (54.4 wt%)
121-8	ACMO (3.2 wt%)	DEGDA (42.4 wt%)	TMP3EOTA (54.4 wt%)
122-8	ACMO (3.2 wt%)	DEGDA (42.4 wt%)	TMPTA (54.4 wt%)
123-8	ACMO (3.2 wt%)	DEGDA (42.4 wt%)	PPTTA (54.4 wt%)
131-8	ACMO (3.2 wt%)	TPGDA (42.4 wt%)	TMP3EOTA (54.4 wt%)
132-8	ACMO (3.2 wt%)	TPGDA (42.4 wt%)	TMPTA (54.4 wt%)
133-8	ACMO (3.2 wt%)	TPGDA (42.4 wt%)	PPTTA (54.4 wt%)
211-8	PHEA (3.2 wt%)	HDDA (42.4 wt%)	TMP3EOTA (54.4 wt%)
212-8	PHEA (3.2 wt%)	HDDA (42.4 wt%)	TMPTA (54.4 wt%)
213-8	PHEA (3.2 wt%)	HDDA (42.4 wt%)	PPTTA (54.4 wt%)
221-8	PHEA (3.2 wt%)	DEGDA (42.4 wt%)	TMP3EOTA (54.4 wt%)
222-8	PHEA (3.2 wt%)	DEGDA (42.4 wt%)	TMPTA (54.4 wt%)
223-8	PHEA (3.2 wt%)	DEGDA (42.4 wt%)	PPTTA (54.4 wt%)
231-8	PHEA (3.2 wt%)	TPGDA (42.4 wt%)	TMP3EOTA (54.4 wt%)
232-8	PHEA (3.2 wt%)	TPGDA (42.4 wt%)	TMPTA (54.4 wt%)
233-8	PHEA (3.2 wt%)	TPGDA (42.4 wt%)	PPTTA (54.4 wt%)
311-8	CTFA (3.2 wt%)	HDDA (42.4 wt%)	TMP3EOTA (54.4 wt%)
312-8	CTFA (3.2 wt%)	HDDA (42.4 wt%)	TMPTA (54.4 wt%)
313-8	CTFA (3.2 wt%)	HDDA (42.4 wt%)	PPTTA (54.4 wt%)
321-8	CTFA (3.2 wt%)	DEGDA (42.4 wt%)	TMP3EOTA (54.4 wt%)
322-8	CTFA (3.2 wt%)	DEGDA (42.4 wt%)	TMPTA (54.4 wt%)
323-8	CTFA (3.2 wt%)	DEGDA (42.4 wt%)	PPTTA (54.4 wt%)
331-8	CTFA (3.2 wt%)	TPGDA (42.4 wt%)	TMP3EOTA (54.4 wt%)
332-8	CTFA (3.2 wt%)	TPGDA (42.4 wt%)	TMPTA (54.4 wt%)
333-8	CTFA (3.2 wt%)	TPGDA (42.4 wt%)	PPTTA (54.4 wt%)
